# Living with multiple sclerosis: A qualitative exploration of death, dying and suicide, in UK adults

**DOI:** 10.1177/13591053251354884

**Published:** 2025-08-25

**Authors:** Daniel Dowling, Lydia Poole, Kimberley J Smith

**Affiliations:** University of Surrey, UK

**Keywords:** death, dying, multiple sclerosis, qualitative, suicide

## Abstract

This qualitative study explored factors contributing to suicidal ideation among people living with Multiple Sclerosis (MS) using the Integrated Motivational-Volitional Model (IMV) of suicide as a theoretical framework. Sixteen participants were interviewed and data analysed using Framework Analysis. The analysis generated four core themes: Theme 1:’Journey to Diagnosis’, captured the difficulties faced by people with MS as they sought a diagnosis and how this could be a trigger point for suicidal ideation. Theme 2: ‘Loss and Deprivation’, captured the struggles and consequences of living with MS which was linked to the transition from the pre-motivational to motivational phase of the IMV. Theme 3: ‘Permanence’, captured the cyclical and chronic nature of MS and was linked to entrapment in the IMV. Finally, theme 4: ‘Death and Suicide’, captured perspectives on death, dying, and suicide and the factors linked to suicidal ideation.

## Introduction

Multiple Sclerosis (MS) is a chronic, progressive, neurodegenerative condition affecting the central nervous system; it affects an estimated 2.3 million people worldwide (Mellentin et al., 2018) and is characterised by the deterioration of the myelin sheath resulting in loss of motor, cognitive, affective and sensory function (Mellentin et al., 2018). MS is linked to physical health problems such as arthritis, irritable bowel syndrome and increased risk of fractures (Marrie and Hanwell, 2013) and mental health difficulties such as anxiety and depression (Feinstein et al., 2014; Janssens et al., 2003). There is also evidence showing that MS is linked to a greater risk of suicide (Shen et al., 2019).

Suicide refers to a fatal, self-injurious act associated with at least some intention to die (Kalb et al., 2019). Suicidal ideation (SI) reflects thoughts to harm oneself or end one’s own life, directly or indirectly or thoughts of being better off dead (Gaskill et al., 2011). Current estimates suggest the lifetime prevalence of suicide attempts to be 4.1% (D’Andrade et al., 2023) and suicidal ideation to be 13% (Kouchaki et al., 2020) amongst people living with MS (PLwMS). Indeed, studies estimate suicidal behaviour to be 2–7.5 times more likely in PLwMS compared to the general population (Sariaslani et al., 2021).

Gaskill et al. (2011) posit that suicide may offer a form of control to PLwMS in the face of an unpredictable disease. Their qualitative analysis highlighted themes around a perceived loss of control, loneliness, physical and psychological impacts of MS and failure or anticipated failure to live up to expected functioning. Other risk factors identified include depression, substance use, social isolation, loneliness, older age, functional limitations, deteriorating condition, pain, fatigue and lack of support (Feinstein, 2002; Kalb et al., 2019; Viner et al., 2014). These risk factors span both generic and MS-specific risks, suggesting that a more nuanced understanding of what contributes to suicidal ideation and behaviour in PLwMS is required.

The Integrated Motivational-Volitional (IMV) model of suicide (O’Connor, 2011; O’Connor and Kirtley, 2018) synthesises components of earlier theoretical models of suicide into a single framework. It aims not only to identify factors which lead to suicidal ideation, but also the factors which bridge the gap between intention and action. The IMV is a three-stage model comprising a pre-motivational phase, motivational phase and volitional phase. The pre-motivational phase is based on a diathesis-stress model in which various biopsychosocial vulnerabilities contribute to an increased risk of suicidal ideation in response to stressors. The second phase suggests that feelings of defeat and/or humiliation, in the context of certain moderators (e.g. negative thinking styles), lead to feelings of entrapment, which in the context of further moderators (e.g. thwarted belongingness and perceived burdensomeness), lead to suicidal ideation. Defeat is described as the feeling that one has been knocked back, while entrapment expresses the prospect that the future holds no escape or rescue (O’Connor et al., 2016). The literature further distinguishes internal and external components of entrapment. Internal entrapment refers to the perceived inability to change an undesirable aspect of oneself, while external entrapment is defined as the sense that one is unable to escape externally derived, undesirable life circumstances (Cramer et al., 2019). The final phase of the model outlines the factors that bridge the gap between intention and action, with the transition being facilitated by factors such as planning, previous exposure to suicide, access to means and past suicidal behaviour. There is some support for the application of the IMV (e.g. Wetherall et al., 2018) but there is a need to further explore the application of this model in specific populations.

To date, there is a lack of research exploring the perceptions towards suicide within PLwMS and the pathways which may lead to suicidal ideation and suicidal behaviour. Prior research has not been theoretically driven, failing to apply a framework of suicide, such as the IMV, to guide understanding of this behaviour. As such, the aims of the current study were to explore PLwMS’ experiences of living with MS and their thoughts surrounding death, dying, and suicide, using the IMV model of suicide as a conceptual guide.

## Method

### Patient and public involvement (PPI)

Due to the sensitive nature of this topic, we consulted with members of the University of Surrey’s Service Users and Carers Advisory Group and MS society on the aims of the study, advertisement of the study, how best to approach the topic of conversation and the interview schedule (wording and questions). The wording of the interview schedule questions was discussed with both groups and refined accordingly.

### Design

This was a qualitative cross-sectional study using a semi-structured interviewing process with PLwMS. Interviews were conducted face-to-face or online according to participant preference. This paper has been drafted in accordance with the COREQ (COnsolidated criteria for REporting Qualitative research) checklist; see Supplemental File 1.

### Participants

Purposive sampling was employed to recruit participants. Inclusion criteria were being 18 years or older with a diagnosis of MS and the ability to speak English. Exclusion criteria included being in receipt of a diagnosis of a comorbid life-limiting condition (e.g. cancer), dementia, brain injury or learning disability, and/or being in receipt of crisis care. These exclusion criteria were selected to avoid confounding of MS-specific findings and to exclude those without capacity to provide informed consent.

We aimed to recruit up to 20 participants in line with Braun and Clarke’s (2013) recommendations. Following transcription and analysis of 16 interviews no new information emerged and recruitment was ceased.

Participants were recruited with the help of voluntary sector groups, such as the MS Register and the MS Trust. Seventy-three potential participants responded to these groups’ advertisement via social media, contacting the researcher via email to express initial interest in the study. Of those, 57 did not respond or return the consent form. The remaining 16 participants were interviewed.

### Semi-structured interview

The interview schedule was informed by the IMV and PPI consultations. To broach the topic of death and suicide, we asked initial probing questions regarding general experiences of difficulties since diagnosis (‘*since diagnosis, have you had any thoughts regarding being better off dead or of death’)*, before asking more general questions linked to death and dying and then suicide itself. The interview schedule is available in Supplemental File 2.

All participants took part in a single 1:1 interview. Most of the interviews took place via video conferencing (13/16) with three being conducted face-to-face in participants’ homes. All interviews were conducted by the lead researcher (DD), a male trainee clinical psychologist who had no prior relationship with any of the participants. Participants were aware this project was part of the lead researcher’s clinical training, and that they held an interest in health psychology and suicide research. Interviews lasted between 30 and 95 minutes (*M* = 64.44, SD = 18.06). All interviews were audio recorded, transcribed verbatim with the help of otter.ai online software and checked for accuracy by DD. Although available by participant request, no transcripts were not returned to participants for comment and/or correction. Field notes were taken by DD to be referred to during data analysis.

### Analysis

Interview data was analysed using Framework Analysis (Ritchie and Spencer, 1994) which is comprised of five stages: familiarisation, identifying a framework, indexing, charting and mapping and interpretation (Parkinson et al., 2016; Ritchie and Spencer, 1994). Framework Analysis can be shaped by existing ideas and theories, without the need to generate a novel theory, allowing for both a deductive and inductive approach (Ward et al., 2013). Coding and charting were led by DD and refined through discussion with LP and KS. Data are reported as quotes followed by a pseudonym and age in years in parentheses.

## Results

Participants were aged between 26 and 68 years (*M* = 37.44, SD = 11.14), predominantly female and White British. Twelve participants had a diagnosis of relapse-remitting MS and four had a diagnosis of secondary progressive MS. Half of participants reported experiencing suicidal ideation since the time of diagnosis. Of these, four disclosed suicidal ideations prior to diagnosis, while one participant noted suicidal ideation prior to diagnosis, but no instances since being diagnosed. See Table 1 for participant characteristics.

**Table 1.** Participant demographics.

Interview ID	Pseudonym	Gender	Age (years)	Ethnicity	MS type	Suicidal Ideation Pre Dx	Suicidal ideation Post Dx
1	Mike	Male	45–50	White British	SP	No	Yes
2	Elisabeth	Female	55–60	White British	SP	No	No
3	Sam	Male	30–35	Black British	SP	No	Yes
4	Symone	Female	35–40	Black British	SP	No	Yes
5	Susan	Female	55–60	White British	RR	Yes	Yes
6	Robyn	Female	40–45	White British	RR	No	No
7	Alexis	Female	25–30	Black British	RR	No	No
8	Izzy	Female	25–30	Hispanic	RR	Yes	No
9	Emma	Female	30–35	White British	RR	No	No
10	Sophie	Female	25–30	White British	RR	No	Yes
11	Sally	Female	35–40	White British	RR	Yes	Yes
12	Megan	Female	25–30	White British Thai	RR	No	No
13	Roland	Male	30–35	White British	RR	Yes	Yes
14	Jamie	Female	25–30	Asian British	RR	No	No
15	Annie	Female	35–40	White British	RR	Yes	Yes
16	John	Male	50–55	White British	RR	No	No

Information regarding suicidal ideation pre- and post-diagnosis was ascertained through analysis of transcripts.

Dx: diagnosis; SP: secondary progressive; RR: relapsing-remitting

Framework Analysis of the 16 semi-structured interviews produced four core themes: (1) Journey to Diagnosis; (2) Loss and Deprivation; (3) Permanence; and (4) Death and Suicide (see Table 2). These themes capture the aspects of people’s experiences, specific to MS, that led them through phases of the IMV and perpetuated thoughts of death and dying, including suicidal ideation.

**Table 2.** Themes and related Sub-themes.

Themes	Subthemes
**Journey to Diagnosis -** a *lot of going to see my GP or going to see my GP practice getting basically ignored and gaslighted (Izzy, 25-30)*.	
**Loss & Deprivation -** *The MS has deprived me, actually deprived is quite good word, of my life. . . . . . It’s deprived my life, it’s deprived me. . . . . . It stopped me from just living normally. I’m very resentful of MS (Susan, 55-60).*	A Daily Struggle Control Disability & Identity
**Permanence -** *Normally you get an illness, and it goes. But this is here to stay, and that. . .that permanence has shocked me a bit, shocked me. (Mike, 45-50)*	
**Death and Suicide -** There is still a stigma about suicide, massive, and everyone assumes, well a lot of people still assume it’s a cop out. . . they don’t actually acknowledge why or how somebody has got to that point. . . . . .whether its illness invoked, or whether it’s pure mental health. . . . . . that’s the bit that people won’t talk about. (Susan, 55-60)	MS and Death. MS and Suicide Assisted Suicide – *A one way ticket to Switzerland*

### Journey to diagnosis

Receiving an MS diagnosis was described as a challenging and often traumatic life event that can be viewed as a potential trigger point in the pre-motivational phase of the IMV. Many participants spoke of the uncertainty and fear at the onset of symptoms, describing a tiresome, prolonged, complicated and painful journey to diagnosis. Most participants gave negative accounts of these early interactions with healthcare services, with reports laced with frustration, fear, distress and shame.

A resounding feature of these experiences was not being believed by healthcare professionals (HCPs) that something was wrong.

I went to a GP and he said, “Oh you know, our minds can play tricks on us sometimes”…… I just thought ‘no’, I’m gonna go back to my own GP…… and she referred me to a neurologist. Even she said, I don’t think there’s anything to worry about. (Robyn, 40–45)

This lack of belief was often paired with poor communication and emotionally distant interpersonal styles that lacked empathy and sensitivity, which persisted into secondary care settings particularly around delivery of diagnosis. Many participants were left feeling dismissed and let down by their encounters with HCPs. For some, these encounters were directly attributed to the onset of suicidal thoughts:

My diagnosis journey was not straightforward at all……that experience of being that young and being told, in lots of roundabout ways that it was looking really serious. That’s what quite often drove me to feeling suicidal (Sophie, 25–30).

Some participants disclosed suicidal ideation and behaviour prior to their diagnosis, referencing a combination of factors including a history of mental ill-health, life stressors and lack of control, all of which may result in additional vulnerability to suicidal ideation and behaviour post-diagnosis.

### Loss and deprivation

This theme represents the daily struggles and consequences of living with MS that contributed to a sense of defeat, humiliation and entrapment, representing a transition from the pre-motivational to motivational phase of the IMV. Participants often framed consequences in terms of a loss. A loss of physical ability, a loss of interest – in hobbies and activities, a loss of independence, a loss of work.

#### A daily struggle

Nearly all participants recognised an impact, to varying degrees, of living with MS on their day-to-day lives, reaching into even the most basic aspects of daily living. From debilitating symptoms including fatigue, mobility issues, cognitive and sensory difficulties, neuropathic pain, incontinence and communication difficulties, to consequences such as challenges to physical functioning and difficulties at work. Notably, these consequences came with an emotional impact. Feelings ranged from anger and frustration to sadness, shame, stress and anxiety. For some participants, it was the combination of repeated stressors that mounted pressure and led to distress and a struggle to thrive in the face of a progressive disease. The degree of difficulty with even the most mundane of tasks and basic bodily functions conveyed a violation of dignity and a sense of humiliation for some participants.

…… every day is a daily struggle. Each day is hard. So little things, like going for a wee, is hard…… it’s little things like that…… all these little things top up, and by the end of the day you’re knackered. So even just going for a wee, having a shower, you go to the toilet, s**t yourself…the little things like that add up, add up, add up, and you just think what’s the point, it’s horrible, this is my day-to-day life. (Mike, 45–50).

Perceptions of loss, failure and a sense of powerlessness over these physical effects of MS and the resulting emotional impact were viewed to contribute to defeat:

. . …my daughter is 15 and she will often say, do you want to go to the cinema, but my daughter doesn’t want us all to go together, because I make things slower …… I feel defeated when my wife and daughter will do things together and I can’t go (Mike, 45–50)

This in turn was considered to contribute to feelings such as depression and anxiety. In Symone’s case, the inability to escape her diagnosis (internal entrapment), coupled with the consequences of her disease leading to potentially undesirable circumstances from which she could not escape (external entrapment) contributed to the development of suicidal ideation:

It was the fear of not being measured up with my equals…… a fear of knowing that these ailments may not allow me to get married…… Those fears grip into me and it’s next thing I want to do something stupid [ending her life]. (Symone, 35–40)

In contrast, despite having the same relapse-remitting diagnosis as others, for some participants MS had a minimal impact on their physical functioning, which seemed to mitigate feelings of defeat and entrapment:

Yeah, I think I just feel quite lucky…… I think because there isn’t that physical……I haven’t got a problem with anything else. I could forget that I’ve got it. (Robyn, 40–45).

#### Control

Discussions on the unpredictable nature of MS were associated with feelings of anxiety and uncertainty among many participants. Participants indicated this uncertainty left them feeling helpless and lacking control, whether over bodily functions, their symptoms or their future.

Again, it’s the helplessness, I’m realising I’ve quite an issue with that…… yeah not being… a burden on somebody else. The lack of control for myself (Roland, 30–35).

Some participants spoke of seeking control in reference to the treatment and management of their MS through things such as lifestyle changes. However, the unpredictable nature of MS could be seen to thwart attempts of control and coping, leading to setbacks and failures. This may have enhanced feelings of defeat and entrapment, leaving people feeling powerless with little to no alternatives to escape their diagnosis or the subsequent circumstances arising because of their MS.

I did everything. I did absolutely everything. …… I exercise as much as I can, I go to therapy, I get fresh air, I get eight hours of sleep a night. I’ve quit caffeine, I’ve quit alcohol. I’ve quit smoking, I’ve quit drugs. What is left? Turns out there were a few things left. …… I do worry, sometimes ……What if I actually exhaust every option? (Sophie, 25–30).

On the other hand, some individuals felt in control of their MS and expressed confidence in the ability of treatment to manage their MS, which was considered to act as a buffer to feelings of defeat and entrapment.

I’m now on my fifth DMT disease modifying therapy to drugs……. I actively pursue the most effective treatment, ……. Just try and pursue the most active treatment because you are preventing future risk. You’re getting a better seat belt. (John, 50–55).

#### Disability and identity

Many participants spoke of the impact of MS on their sense of self, speaking of changes to and a loss of identity. At times there seemed to be a conflict between the relief of explaining difficulties through the label of MS, and the frustration of being defined by it. For Susan, she described the bewilderment of having to learn how to create a new sense of self, to incorporate her MS:

Just everything that could go wrong is going wrong. And my biggest issue is around grieving for the loss of my old me. Because I look like me, but I’m not me…… I’ve lost my identity. For 50 years, I knew exactly who I was. And now I haven’t got a clue who I am (Susan, 55–60).

For many participants, their change of, and loss of identity was linked to disability. For a number, the label of disability conflicted with their sense of self, particularly if the disability was not visible.

I got in the post from the County Council, a little yellow card that says you’re officially registered as disabled…… I really don’t feel like I am at all…… I’ve just run a 5k at the gym did some weights. I really wouldn’t class myself as that, but other people do. (Robyn, 40–45)

Several participants expressed fear and concern at the prospect of a slow and gradual decline in functioning, as well as worries for the stigma that may come with it. This fear of increasing disability and the inability to recover could entrap participants.

The degree of disability described varied across participants and was closely linked with the extent to which negative views were expressed, suggesting that the degree and impact of disability may influence the levels of defeat and entrapment experienced by participants. For some, increasing disability came with a fear of increased burden on others, and defeatist thoughts.

If I’ve gone from how I am now to wheelchair bound and needing a team of carers, then I think my mood would be so low that…… What’s the point? What am I living for? (Roland, 30–35)

### Permanence

This theme represents the cyclical nature and chronic timeline of MS. The permanence of MS embodies the concept of entrapment, in particular internal entrapment, whereby individuals have a desire to escape the illness threat but are blocked in doing so by the nature of the disease. This permanence can bring a pessimism for the future and foster ruminative thoughts that things are not going to get better. For Sophie, it was this permanence of MS and the inability to escape her diagnosis, the internal entrapment, that contributed to her suicidal ideation.

I was actually with the love of my life, and everything was so good, and then all of a sudden, the future was this horrible thing, and that was when it happened, I was like shit, I feel suicidal……. I felt suicidal when I found out it was going to be a forever thing. (Sophie, 25–30).

Permanence was also seen to compound feelings of lack of control and disability. MS is unpredictable by nature, and participants struggled to picture what their future would look like, though most included images of disability. One participant expressed a preference for a terminal diagnosis, as at least then they could form a clear and coherent timeline of their future. At the same time, participant’s spoke about the resilience they had developed from living with MS,; as Sophie explains, MS is a *‘double-edged sword’* that on the one hand could be defeating and on the other empowering:

dealing with this was a double-edged sword because like I said, it added to that hopelessness feeling ……. But on the other hand…… it’s also been like, you’ve been doing this for so long, and you’re still here…… like you’ve done it before you can do it again. (Sophie, 25–30)

### Death and suicide

This theme represents participants’ perspectives on death, dying and suicide in the context of living with MS, and motivational factors which may directly influence the formation of suicidal ideation.

#### MS and death

Among participants there was a general sense of acceptance around death, with many seeing it as a natural and inevitable part of life. Some expressed feeling too young to have to think about death or had not been exposed to death more broadly in their lives but felt that MS had made them confront their mortality. Within these discussions several participants expressed contrasting views on the association between MS and death, highlighting possible differences in understanding and meaning making. For some MS and death were fundamentally synonymous.

Although MS is not classified as a terminal illness, it will kill you. You know, ultimately, you’re going to get a massive infection, you’re going to get complications……you’re going to get something and it’s going to kill you, and you can directly link it back to the MS. (Susan, 55–60)

Here Susan equates MS to an almost inevitable death sentence in her sense-making. Such a strong association with mortality may heighten perceptions of threat and fear, yielding higher levels of distress and difficulty in adjusting to the disease psychologically. At the same time, this view may lead to a desire for a hastened death, bridging the gap between intention and action.

I’d describe myself as being on borrowed time, now. I believe that I’m on a very shortened lifespan … which will almost certainly be of my choice rather than necessarily my body of choice if you know what I mean (i.e., suicide). (Susan, 55–60)

For others, there was a clear distinction between MS and death. For Sally, MS wasn’t perceived to be terminal.

I’d say it’s completely separate …… I don’t even think about them in the same breath…… I’m very aware that lots of things can kill people, it’s very rarely MS. MS doesn’t kill people. (Sally, 35–40).

John also felt that MS and death were separate and expressed concern that people’s belief about the association between MS and death may influence their decisions around the necessity and urgency of treatment as well as tolerance of medication side effects. These treatment beliefs could, in turn, have ramifications for illness outcomes such as physical and cognitive functioning as well as emotional adjustment.

MS and death are not associated. If I said to you cancer……. cancer and death are just intermeshed. So, that means then that goes to the risks people are prepared to take for treatment, because they can suddenly see a far more immediate threat to life. (John, 50–55)

### MS and suicide

Half of the participants in this study disclosed experiencing suicidal ideation since their diagnosis. Narratives around contributing factors included aspects that were separate to or difficult to disentangle from MS. Some participants spoke of the distress associated with diagnosis and the permanence of MS:

At that initial stage…I didn’t understand myself, so I thought of leaving the world…….it was my fear of living with such a condition that makes me thought of leaving the world. (Symone, 35–40).

While others touched on aspects of defeat, thwarted belonging and perceived burdensomeness:

I feel like I am in the way of my family, and I feel if I wasn’t here, it would be so much easier for my wife, my daughter, so much easier…… (Mike, 45–50)

Some participants described the potential plans to end their life that had formed in their minds, with varying degrees of lethality, speed and associated pain:

I actually thought of drowning myself and I sometimes thought of, you know, hanging myself and taking some kind of poisonous substance… (Sam, 30–35)

However, none reported making an attempt on their life since diagnosis. Participants cited factors such as family, religion, adaptation, social support and flexible thinking as protective factors.

As time goes on, as I live, I begin to see myself adapting to the situation, adapting to my new kind of life and moving on…… Like my mom will always say God will send an angel (Symone, 35–40).

### Assisted suicide – A one-way ticket to Switzerland

Just under half of the participants referenced discussions on the topic of assisted suicide. Core elements of these discussions, which may contribute to the development of suicidal ideation or drive a transition from the motivational to volitional phase of the IMV, centred on a severe loss of independence, deteriorating quality of life and perceived burdensomeness on loved ones.

When I have had those, those low points I’ve always had a long running joke but with it with an element of seriousness with it, of saying to one of my best mates and to my mum, my sister, if something goes really wrong, I want a one-way ticket to Switzerland. (Roland, 30–35)

Some participants viewed assisted suicide as a valid option in the context of *‘existing as opposed to living’* and voiced a desire to be able to die with dignity. Challenges in making an informed decision were recognised due to the lack of clarity regarding prognosis, and the unpredictability of the disease. For some these views were held prior to their diagnosis of MS and extended to other contexts.

I’ve always been of the mindset that if I was to end up in a position where I was what I would call existing as opposed to living. If I have no independence, and I was relying on someone else to take care of me and every aspect, I would consider suicide as an option. (Emma, 30–35)

## Discussion

**Figure 1.**
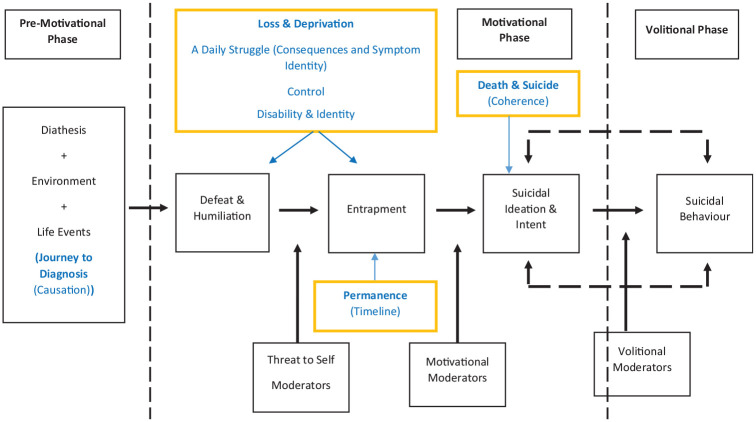
Proposed incorporation of findings in context of IMV.

While exploring people’s experiences of living with MS, viewing our data through the framework of the IMV, we heard varying accounts of defeat, entrapment and suicidal ideation. Aspects of people’s experiences, specific to MS, that directed them through phases of the IMV, centred around the themes of ‘Journey to Diagnosis’, ‘Loss and Deprivation’, ‘Permanence’ and elements of ‘Death and Suicide’. Figure 1 shows a proposed depiction of the relationships identified through this study, incorporated with the IMV.

Participant’s journey to diagnosis stood out as a significant life event, which could be mapped to the pre-motivational phase of the model (see Figure 1). Our findings support previous studies looking at the experience of diagnosis for PLwMS which have yielded reports of vulnerability, communication problems and lack of support, with common emotions including fear, anxiety, shame, and sadness, with preconceptions of MS laced with disability and death, and for some thoughts of suicide (Edwards et al., 2008; Fallahi-Khoshknab et al., 2014). An individual’s diagnostic journey has the potential to exacerbate pre-existing mental health difficulties, lead to emotional distress and shape negative cognitive appraisals (Topcu et al., 2020), all of which can contribute to the development of suicidal ideation according to the IMV.

While exploring perceptions of defeat and entrapment among PlwMS, participants often referenced different aspects of their illness, which can be seen to align with elements of the CSM (Leventhal, 1970; Leventhal et al., 1980, 1997). According to the CSM, individuals develop a set of beliefs regarding their illness, which are then used to guide decisions regarding self-management of the disease, and ultimately impact illness outcomes. Illness perceptions include the label attached to the disease and its symptoms (Identity), beliefs regarding its determinants (Causation), progression and duration (Timeline), perceptions of the impact on day-to-day functioning (Consequences) and level of perceived control over the illness (Control), as well as understanding of the illness (Coherence; Luca et al., 2022). Several of these beliefs were seen to be represented amongst our themes and could also be mapped to the motivational phase of the IMV.

A recurrent feature within participant’s experiences of ‘A Daily Struggle’ was the description of the limiting symptoms and negative consequences that come with living with MS. Research into MS and illness perceptions has shown that a greater number of symptoms and perception of more severe consequences is often related to worse outcomes, including higher levels of depression and anxiety and greater impairment in physical functioning (Luca et al., 2022; Vaughan et al., 2003). It is possible that a greater number of symptoms and the associated negative outcomes may increasingly lead to feelings of defeat, with certain symptoms bringing feelings of embarrassment or humiliation. As individuals suffer continued loss and become to feel more restricted by their condition, they may feel trapped in their situation. In contrast, lower levels of observed symptoms and perceived consequences may mitigate feelings of defeat and entrapment, leaving them more capable of managing stressors and engaging in adaptive coping mechanisms.

Aspects of ‘Control’ centred on participants’ perceptions of control over their lives and MS. Research on illness perceptions indicates that beliefs of uncontrollability have been associated with higher levels of depression and anxiety in a range of illnesses (Capobianco et al., 2020). Additionally, in an MS specific study, Gaskill et al. (2011) found a perceived loss of control to be associated with suicidal ideation. Personal control (beliefs about personal influence over the illness) and treatment control (beliefs about the effectiveness of medical or treatment plans) may be seen to relate to internal entrapment (Bassi et al., 2016). It is possible that individuals with low levels of personal and treatment control may experience more feelings of internal entrapment, as they feel powerless to do anything to escape or change their situation. In contrast, high levels of control may act as a protective factor, offering individuals hope, allowing for more cognitive flexibility and adaptive coping strategies that reduce feelings of internal entrapment.

The theme of ‘Permanence’ related to the chronic nature of the illness. Within the MS literature, some studies have reported the cyclical and chronic nature of MS to be linked with poorer outcomes such as higher levels of anxiety and fatigue (Bassi et al., 2020, 2021; Jopson and Moss-Morris, 2003), while others state these features are associated with positive outcomes such as adversarial growth (Ackroyd et al., 2011). In their exploration of the impact of chronic illness and suicidality, Karasouli et al. (2014) found all participants, who had either MS or chronic kidney disease, struggled with the chronic and incurable nature of their conditions, describing continuous battles and never-ending challenges. In the context of MS and the IMV, the prospect of yet another battle during a flare and another potential defeat, may reinforce feelings of internal entrapment, in this instance the inability to escape one’s illness, and contribute to the development of suicidal ideation.

‘Disability’ may also be viewed to relate to both internal and external entrapment, as the MS itself or the physical consequences arising from it. Research has previously identified a strong link between disability and depression within MS (Arnett et al., 2008), and recent research has begun to indicate that individuals with a physical disability more generally are at a higher risk of suicide (Khazem et al., 2015), with the continued erosion of people’s independence being identified as a contributing factor (Kennedy et al., 2021).

During our interviews, participants expressed varying views on the topics of death and suicide. A number of participants were accepting of death, seeing it as a natural process and something that is inevitable for us all. Some held a strong belief about the link between MS and death, while others viewed them as independent. Previous research has shown that common perceptions of MS include death for example, Isaksson and Ahlstrom (2006), though there is often a fear of discussing it (Cottrell et al., 2020). Similar to Golla et al. (2016), our analysis shows that there are a variety of perspectives on death and dying within PLwMS that require differentiated approaches to support them.

In discussions around suicide, several participants touched on the topic of ‘assisted suicide’. Lewis et al. (2017) references the term ‘rational’ suicide, recognising that suicide has been observed in the absence of depressive symptoms. Criteria for ‘rational’ suicide include (1) the presence of an unremittingly hopeless condition, (2) a suicidal decision made as a free choice and (3) the presence of an informed decision-making process (Werth and Cobia, 1995). These criteria touch upon points made by our participants in their perspectives on assisted suicide, and the importance of an informed choice that allows them to die with dignity. On the other hand, there are those that argue that the uncertainty and unpredictability regarding the progression of MS, mean making a clear and informed decision is extremely difficult (Coles, 2017). This has implications for the timeline of such a choice for our participants who were based in the UK, whereby the requirement of independent travel to assisted suicide clinics overseas may push an individual to decide prematurely. For now, the debate regarding the ethics and legality of assisted suicide/euthanasia in the UK continues.

### Strengths and limitations

The present study contributes to the growing evidence base by addressing a gap in knowledge by exploring the applicability of the IMV model in the context of MS. A strength of this study was our PPI which allowed us to thoroughly consider ethical issues and ensure sensitive framing of research materials. The application of a Framework Analysis yielded a structured, transparent, and flexible approach, with a step-by-step process allowing for clear replication. Taking a qualitative approach permitted participants to express their experiences, perspectives, beliefs and ideas for a more authentic representation. While this is not the first study exploring PLwMS’ perspectives on death, dying and suicide, it is the first that considers these in the context of the IMV.

However, the qualitative nature of this study leaves it vulnerable to the influence and biases of the researcher. In attempt to minimise these limitations, the researcher kept a reflexive diary to reflect on personal experiences and emotions, better understand their assumptions and aid transparency. Furthermore, half of the sample had experienced suicidal ideation which could have implications for the themes that were generated. The presence of pre-diagnosis suicidal ideation suggests that for some individuals, MS may have intersected with an existing vulnerability rather than being the sole or initiating factor, which could influence both the nature and persistence of distress. Future research should explore this distinction further.

### Implications for research and clinical practice

Models of suicide continue to be researched and developed so that the evidence base can be incorporated to aid the identification of those at risk of suicidal ideation and behaviour and develop targeted interventions to prevent suicide. Findings from this study call for the consideration of the role of illness perceptions in the development of suicidal ideation in the context of MS. A better understanding of this relationship could potentially identify avenues for treatment that is, through interventions targeting illness perceptions. Research may also look to expand the investigation of illness perceptions within the IMV to other chronic conditions.

A recurring point arising from participants’ narratives was a loss of control. Interventions targeting specific illness perceptions such as coherence and control, by increasing understanding of their illness and promoting autonomy through shared decision-making and supported self-management programmes may help individuals regain a sense of control and improve outcomes, which may reduce feelings of defeat.

While the impact of MS subtype was not explicitly explored during interviews, from the perspective of the IMV model, the loss of treatment options and anticipated functional declined associated with the transition from relapse-remitting to secondary-progressive MS could plausibly contribute to key drivers of suicidal ideation and behaviour. Thus, an exploration of the interplay between clinical MS-specific factors for example, subtype and other psychosocial risk factors may prove valuable.

The theme of Loss and Deprivation captured participants’ expressions of perceived burdensomeness. As perceived burdensomeness is often a distorted perception, one which has repeatedly been linked with suicide, this may act as a plausible target for intervention. Lieberman et al. (2023) demonstrated that therapeutic interventions such as Collaborative Assessment and Management of Suicidality, as well as Social Problem-Solving Treatment effectively reduced suicidal ideation amongst veterans, citing the mediating role of targeting perceived burdensomeness.

It should be noted that this study’s findings relate mostly to the motivational phase of the model. While motivational variables have been useful to help identify those at risk of suicide, they are not key drivers in enaction and there is a concern that they do not distinguish ideators from actors (Wetherall et al., 2018). As such, further investigation of the volitional phase is warranted to address this gap.

A notable finding centred on the experience of participants’ journey to diagnosis and the poor rapport with HCPs. Poor communication between HCPs and patients can result in negative experiences of care making PLwMS feel unsupported (Johnson, 2003; Malcomson et al., 2008). There is a need for more sensitive person-centred support and advice for PLwMS at time of diagnosis. Soundy et al. (2016) outline possible solutions which include enhancing communication by reducing clinic jargon, avoiding dismissive attitudes, further training to refine listening and empathy skills, as well as learning therapeutic techniques (e.g. motivational interviewing). Suicidal ideation may not be disclosed unless explicitly and sensitively explored, thus, the development of these skills could aid the screening of suicidal ideation in a direct yet sensitive manner.

## Conclusion

This study offers novel insights into how PLwMS may experience suicidal ideation, using the IMV model of suicide as a guiding framework. Key findings highlight how the diagnostic process, illness perceptions and the chronic, disabling nature of MS can contribute to feelings of defeat, entrapment, and ultimately suicidal ideation. Themes such as *Journey to Diagnosis, Loss and Deprivation*, and *Permanence* mapped closely onto phases of the IMV model, particularly the motivational phase. Illness perceptions emerged as significant psychological mechanisms contributing to emotional distress and suicidal thoughts. The experience of poor communication with HCPs was also found to exacerbate distress, pointing to a critical area for intervention. Future research should test targeted interventions informed by these insights to support suicide prevention in PLwMS.

## Supplemental Material

sj-docx-1-hpq-10.1177_13591053251354884 – Supplemental material for Living with multiple sclerosis: A qualitative exploration of death, dying and suicide, in UK adults Supplemental material, sj-docx-1-hpq-10.1177_13591053251354884 for Living with multiple sclerosis: A qualitative exploration of death, dying and suicide, in UK adults by Daniel Dowling, Lydia Poole and Kimberley J Smith in Journal of Health Psychology

sj-docx-2-hpq-10.1177_13591053251354884 – Supplemental material for Living with multiple sclerosis: A qualitative exploration of death, dying and suicide, in UK adults Supplemental material, sj-docx-2-hpq-10.1177_13591053251354884 for Living with multiple sclerosis: A qualitative exploration of death, dying and suicide, in UK adults by Daniel Dowling, Lydia Poole and Kimberley J Smith in Journal of Health Psychology

sj-pdf-3-hpq-10.1177_13591053251354884 – Supplemental material for Living with multiple sclerosis: A qualitative exploration of death, dying and suicide, in UK adults Supplemental material, sj-pdf-3-hpq-10.1177_13591053251354884 for Living with multiple sclerosis: A qualitative exploration of death, dying and suicide, in UK adults by Daniel Dowling, Lydia Poole and Kimberley J Smith in Journal of Health Psychology
