# Supplementary figures and images for: Living with multiple sclerosis: A qualitative exploration of death, dying and suicide, in UK adults
Source: J Health Psychol. 2025 Aug 25;31(4):1627–42. doi: 10.1177/13591053251354884 (PMC12960738; doi:10.1177/13591053251354884)

**Supplementary file 3: How analysis mapped onto IMV framework**


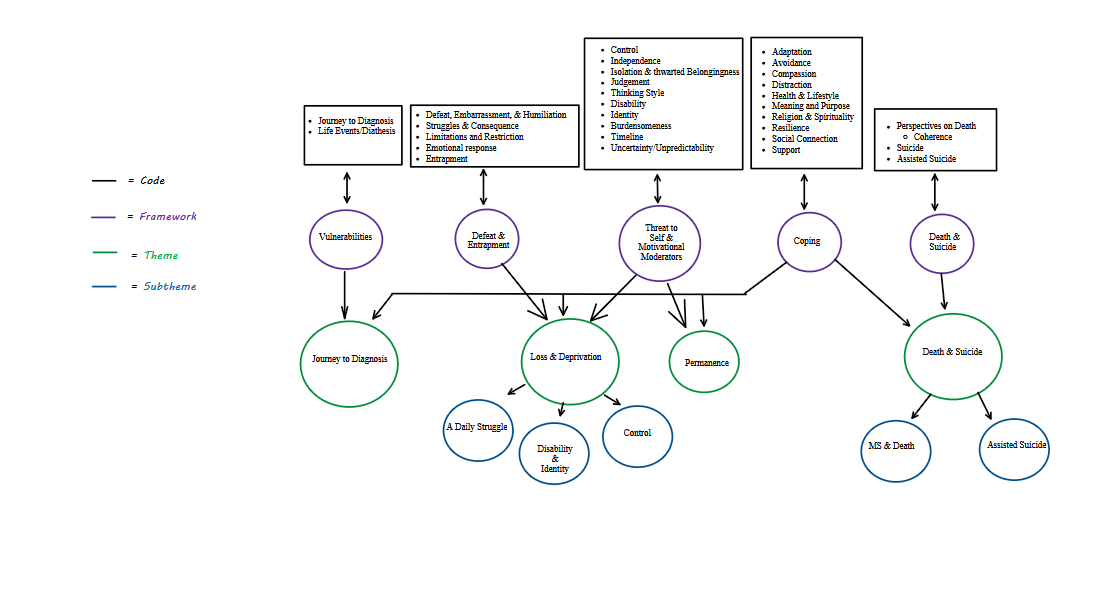

Supplement: sj-docx-1-hpq-10.1177_13591053251354884 – Supplemental material for Living with multiple sclerosis: A qualitative exploration of death, dying and suicide, in UK adults [file sj-docx-1-hpq-10.1177_13591053251354884.docx]
