# Supplementary material for: Living with multiple sclerosis: A qualitative exploration of death, dying and suicide, in UK adults
Source: J Health Psychol. 2025 Aug 25;31(4):1627–42. doi: 10.1177/13591053251354884 (PMC12960738; doi:10.1177/13591053251354884)
Supplement: sj-docx-2-hpq-10.1177_13591053251354884 – Supplemental material for Living with multiple sclerosis: A qualitative exploration of death, dying and suicide, in UK adults [file sj-docx-2-hpq-10.1177_13591053251354884.docx]

## **Supplementary file 2: Interview Schedule**

- - - 1. This interview is to explore the topics of death, dying and suicide amongst people living with MS. Do you have any questions before we begin?
      2. Do you identify any challenges of living with MS/What are some of the main challenges?
      3. Have you felt down, depressed, or anxious since being diagnosed with MS?
      4. Since being diagnosed with MS, have you had any thoughts regarding death or dying?
      5. Have you had any thoughts of being better off dead or of harming yourself in anyway?

| If Yes but no SI | If Yes | If No |
| --- | --- | --- |
| 1. What has been the content/function/meaning of these thoughts? (What form have these thoughts taken? What has shaped these thoughts? How have these thoughts manifested? Why do you think you have these thoughts? Why do you want to end your life? What have been the triggers?) 2. Have you felt comfortable to discuss this in care? | 1. What has been the content/function/meaning of these thoughts? (What form have these thoughts taken? What has shaped these thoughts? How have these thoughts manifested? Why do you think you have these thoughts? Why do you want to end your life? What have been the triggers?) 2. Have you had these thoughts prior to being diagnosed with MS? (if yes, have they changed in anyway since being diagnosed?) 3. What are some of the things that have led you to think about death or dying/feelings that you would be better off dead? 4. In your experience of living with MS, have you ever felt defeated or humiliated by it? 5. In your experience of living with MS have you ever felt trapped by it? 6. How do you cope with the uncertainty/unpredictability of MS? 7. volitional factors:    1. Have your thoughts of death, dying, or ending your life ever formed into plans of how you might act on them?    2. Would you describe yourself as impulsive?    3. What is your tolerance for physical pain like?    4. Are you afraid of death?    5. Have you ever acted on any thoughts to harm yourself or end your own life? | 1. What do you believe may stop you from having these thoughts/feeling like this? |

1. What are some of the things that have helped you adjust to living with MS?
2. Is there anything else you can think of that might add to our understanding of death, dying and suicide for people living with MS, or something you feel you have not had the chance to mention?
3. Overall, how did you feel during this interview/What was it like to be interviewed on this topic?
